# Implementation of Lightweight Convolutional Neural Networks via Layer-Wise Differentiable Compression

**DOI:** 10.3390/s21103464

**Published:** 2021-05-16

**Authors:** Huabin Diao, Yuexing Hao, Shaoyun Xu, Gongyan Li

**Affiliations:** 1 Institute of Microelectronics, Chinese Academy of Sciences, Beijing 100029, China; diaohuabin@ime.ac.cn (H.D.); haoyuexing@ime.ac.cn (Y.H.); ligongyan@ime.ac.cn (G.L.); 2 University of Chinese Academy of Sciences, Beijing 100049, China

**Keywords:** convolutional neural networks, structural compression, differentiable, layer-wise

## Abstract

Convolutional neural networks (CNNs) have achieved significant breakthroughs in various domains, such as natural language processing (NLP), and computer vision. However, performance improvement is often accompanied by large model size and computation costs, which make it not suitable for resource-constrained devices. Consequently, there is an urgent need to compress CNNs, so as to reduce model size and computation costs. This paper proposes a layer-wise differentiable compression (LWDC) algorithm for compressing CNNs structurally. A differentiable selection operator OS is embedded in the model to compress and train the model simultaneously by gradient descent in one go. Instead of pruning parameters from redundant operators by contrast to most of the existing methods, our method replaces the original bulky operators with more lightweight ones directly, which only needs to specify the set of lightweight operators and the regularization factor in advance, rather than the compression rate for each layer. The compressed model produced by our method is generic and does not need any special hardware/software support. Experimental results on CIFAR-10, CIFAR-100 and ImageNet have demonstrated the effectiveness of our method. LWDC obtains more significant compression than state-of-the-art methods in most cases, while having lower performance degradation. The impact of lightweight operators and regularization factor on the compression rate and accuracy also is evaluated.

## 1. Introduction

In recent years, great breakthroughs have been achieved in information retrieval, natural language processing and computer vision due to the performance improvement of CNNs. However, the structure of CNN also becomes more complex, which brings large burdens of storage and computation. This greatly limits their deployment on resource-constrained devices, such as field-programmable gate arrays (FPGAs), digital signal processors (DSPs), cell phones and other mobile devices. Therefore, it is essential to obtain lightweight networks. There is some recent research to get efficient models by designing compact architectures manually, such as SqueezeNet [1], MobileNet [2,3,4] and ShuffleNet [5,6]. Some specific application scenarios of light CNNs also have be proposed, such as Crowd Counting [7], Bone Metastasis Classification [8] and Wheat Head Detection [9]. In addition, searching lightweight architectures automatically by neural architecture search [10,11,12,13,14] has become a research trend recently. In contrast to the aforementioned method of designing compact architectures straightforwardly, compressing the existing CNNs can also derive lightweight models. Current compression algorithms mainly focus on pruning the structural units within the CNNs, including filters, channels and other structural units. These compression algorithms cannot break the limitations of the origin network and can only prune units limitedly under a fixed network structure. In addition, they cannot achieve end-to-end compressing. Such a process is mainly divided into three steps: firstly pre-training the CNN, then removing redundant structural units according to certain criterion and finally re-training the pruned model iteratively. In order to address the aforementioned problems, we propose a new CNN compression algorithm from a novel perspective. Instead of removing units from those redundant convolutional operators, we propose to replace them with more light ones directly, which allows us to break the limitations of original architecture. Our compression algorithm requires initially specifying N lightweight convolutional operators and then using them to reconstruct an over-parameterized CNN with N branches on each layer from a given original CNN. Each branch is multiplied by a trainable mask (whose value can only be 0 or 1), and only one branch among all branches on each layer has a mask of 1. The reconstructed CNN can be trained via gradient descent with a resource-constrained objective function. At the end of training, the branches whose mask is 0 are removed from each layer and thus the lightweight model is constructed by the remaining branches with a mask of 1. Consequently, compact CNNs composed of lightweight operators are derived.

In conclusion, the proposed approach is a layer-wise differentiable compression algorithm. Our main contributions can be summarized as follows:

- The proposed approach addresses the compression problem of CNNs from a fresh perspective, which replaces the original bulky operators with lightweight ones directly instead of pruning units from original redundant operators.
- Most of the existing approaches [15,16,17,18,19] require specifying the compression rate for each layer or require a threshold that is used to determine which structural units to prune. Our proposed approach does not require any such input and can automatically search for the best lightweight operator in each layer to replace the original redundant operator, thereby reducing the number of hyperparameters.
- The proposed approach is end-to-end trainable, which can compress and train CNNs simultaneously using gradient descent in one go. We can obtain various compressed lightweight CNNs with different architectures, which also inspires the future design of CNNs.

The rest of this paper is structured as follows: Section 2 describes the related works in CNN compression. Section 3 presents the proposed methodology. Section 4 describes the experiment in this paper and analyzes the experimental results. In Section 5, the conclusions are given.

## 2. Related Works

In the early stages, compression of CNNs focuses on fine-grained trimming. Although fine-grained compression methods [19] can achieve high pruning rates, the resulting sparse matrices require specialized hardware and software support, making it difficult to obtain actual acceleration. Thereby, the current CNN compression methods mainly focus on coarse-grained trimming, and the pruned units including channels, filters and other structural units. This paper mainly concentrates on coarse-grained compression methods, which include the following categories:

Trimming according to certain criteria: The main process of such pruning algorithms [18,20,21,22,23,24] includes: firstly training a CNN as usual, then pruning units from the trained CNN according to some artificial criteria, finally fine-tuning the slimmed CNN. Li et al. [18] rank the filters based on their norm values in each layer, removing the ones with small norm values. Hu et al. [21] sort the filters according to the ratio of activation values of zero (APoZ) in the feature map, pruning out the ones with larger APoZ values. He et al. [24] argue that norm-based pruning of filters gives better compression results only when the norm deviation of the filter is sufficiently large, so they propose a trimming method based on the geometric median of the filter. Singh et al. [23] order the filters by introducing an auxiliary loss function and evaluating the sensitivity of filter with respect to the auxiliary loss function, pruning out those sensitive ones. The aforementioned methods not only require a pruning criterion designed manually but also need to specify compressing rate, which increases the complexity of algorithm.

Sparse regularization: Sparse regularization algorithms [25,26,27,28,29] realize CNN compression by introducing parameter-related regularization terms in the loss function and thus controlling the number of parameters in the network. Yoon et al. [26] add L(1,2) norms to the parameters at each layer of the network to learn fewer but more useful features to achieve model slimming. Ye et al. [27,29] compress CNNs by introducing the ADMM algorithm to optimize the model under a given parameter constraint.

Low-rank decomposition: Low-rank decomposition algorithms [30,31,32] use a lower-rank set instead of the original set of parameters to approximate the CNN to achieve compression. Swaminathan et al. [31] argue that the low-rank decomposition of weight matrices should consider influence of both input as well as output neurons of a layer. They propose a sparse low rank (SLR) approach that sparsifies SVD matrices to obtain better compression rate by keeping lower rank for unimportant neurons. Ruan et al. [32] construct a compressed-aware block to minimize the rank of the weight matrix and identify the redundant channels automatically.

Automatic pruning algorithm: He et al. [33] use a reinforcement learning method for CNN compression, encoding the compression rate of each layer as the agent’s action, rewarding the agent with validation accuracy, and training the agent so that it can automatically determine the best compression rate used in each layer. Zhu et al. [34] set the compression rate of the model automatically with a heuristic method, however, which needs to determine the target compression rate of the model. Liu et al. [35] combine Alternating Direction Method of Multipliers (ADMM) [36] with the simulated annealing algorithm to automatically prune the network.

Knowledge distillation: Knowledge distillation [37,38,39,40,41,42,43,44] uses a complex CNN model with a large number of parameters to train a network with a small number of parameters to obtain a lightweight network. Wu et al. [42] propose a multi-teacher knowledge distillation framework to compress CNN. Prakosa et al. [43] explore that knowledge distillation can be integrated to pruning methodologies to improve accuracy of the pruned model. Ahmed et al. [44] propose a framework that leverages knowledge distillation and customizable block-wise optimization to learn a lightweight CNN architecture.

Most of the existing compression approaches can only prune a few redundant structural units from the fixed structure of the original network, which results in a low compression rate. In addition, they require specifying how many structural units from each layer to prune, which generates a lot of hyperparameters. In addition, the compression process requires pruning and retraining iteratively, which cannot be done in one go.

## 3. Methodology

### 3.1. Overview

The compression algorithm this paper proposed consists of three stages: the reconstructing stage, the searching stage and the fine-tuning stage. In the reconstructing stage, N lightweight convolutional operators are used to reconstruct an over-parameterized CNN with N branches on each layer from any given original CNN. Each branch multiplies a trainable mask (whose value can only be 0 or 1) and only one branch among all branches on each layer has a mask of 1. The construction of the mask is described in Section 3.3.2. In the searching stage, the reconstructed CNN is trained via gradient descent with a resource-constrained objective function introduced in Section 3.3.3. At the end of training, the branches whose mask is 0 are removed from each layer in this reconstructed CNN, thus a lightweight CNN is constructed by the remaining branches with a mask of 1. In the fine-tuning stage, the lightweight CNN is fine-tuned to obtain a performance improvement.

### 3.2. The Reconstructing Stage

**Figure 1:**
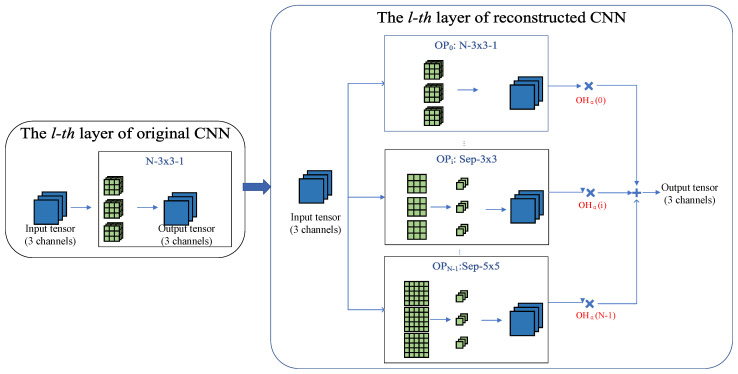
Reconstruction of the original CNN. We expand each layer (convolution layer or pooling layer) into N parallel branches and multiply a trainable mask to each branch. In this figure, $OH_{\alpha }$ is a one-hot mask vector generated by α. Each element of $OH_{\alpha }$ can only be 0 or 1 and only one element can be 1. $OH_{\alpha }$ can be trained using gradient descend. α is defined as architecture parameter. The layer index of α is omitted which should be $\alpha^{l}$ for the lth layer. The blue squares represent input or output tensors (with 3 channels for better presentation) and the green squares represent weights of convolution layers. Only three operators are presented in the figure, and more lightweight operators are shown in Appendix A.

In the reconstructing stage, an over-parameterized CNN with multiple branches on each layer is reconstructed from any given original CNN. The lightweight CNN can be obtained by training this reconstructed CNN. Figure 1 shows one convolutional layer (left) in the original CNN and its corresponding layer (right) in the reconstructed CNN. Each convolutional layer (or pooling layer) is expanded into N parallel branches in the corresponding layer of the reconstructed CNN.

The operators chosen in the branches are lightweight that have fewer parameters and floating-point operations (FLOPs) than the corresponding operators in the original CNN, such as group convolution [5,6], depthwise separable convolution [2,3] and CReLU convolution [37] (replacing ReLU with CReLU can save half of the channels). We experiment not only with these simple lightweight operators but also with other more complex lightweight convolutional modules, such as the Fire module [1], the module with residual connections [45]. We can also combine these different features to form new lightweight operators, e.g., combining the group feature with the CReLU, or the depthwise separable feature. More details are shown in Appendix A, including the detailed structure, the number of parameters, and the FLOPs of these lightweight operators.

The output tensors from multiple branches are integrated into the final output tensor of this layer in the reconstructed CNN. We use weighted sum as the integration strategy and the weights are represented as $OH_{\alpha }\left(\cdot \right)$ in Figure 1. $OH_{\alpha^{l}}$ is a one-hot mask vector generated by $\alpha^{l}$, and its construction is described in detail in Section 3.3.2. *l* is the layer index. The convolutional operator for each branch is denoted as $OP_{i}$, i = 0, 1, …, N − 1. For ease of description, we define the convolutional operator corresponding to the reconstructed layer containing multiple branches as the selection operator OS, and then obtain

(1) $$OS^{l}\left(\cdot \right)=\sum_{i=0}^{N-1}OH_{\alpha^{l}}\left(i\right)\cdot OP_{i}\left(\cdot \right)$$

### 3.3. The Searching Stage

**Figure 2:**
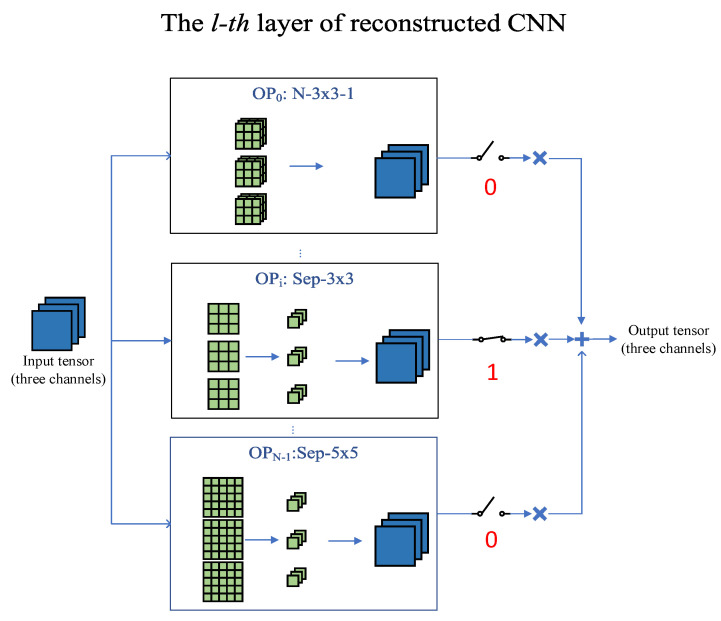
Illustration of the searching stage. In the searching stage, the mask of each branch $OH_{\alpha^{l}}\left(i\right)$ can be simultaneously learned with the parameters of convolutional operators. If $OH_{\alpha^{l}}\left(i\right)=0$ in the current training step, it means the *i*th branch does not work at this moment, and vice versa. The reconstructed CNN is equivalent to the child CNN formed by the branches whose mask is 1 in each training step.

In this section, a trainable gate function is constructed firstly, then a continuous approximation for the discrete one-hot mask vector $OH_{\alpha }$ is performed based on the gate function. Next, the resource-constrained objective function is described and the reconstructed CNN is trained using it. In the searching stage, the value of mask vector $OH_{\alpha }$ is simultaneously learned with the parameters of the convolutional operators of each layer. Additionally, there is only one branch per layer with a mask of 1, and only the branch with a mask of 1 works, as shown in Figure 2.

#### 3.3.1. Trainable Gate Function

The gate function TG(ω) is defined as

(2) $$\mathrm{TG}\left(\omega \right)=\left\{\begin{array}{cc} 1 & \omega >0\\ 0 & \omega \leq 0 \end{array}\right.$$

**Figure 3:**
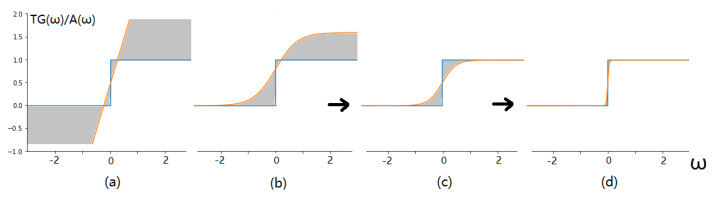
Illustration of the asymptotic approximation function. (**a**) The comparison between the identity approximation and the original function TG(ω), where the gray shaded part indicates the approximation error, indicating a large error in the identity approximation. (**b**–**d**) Different stages of asymptotic approximation, which gradually approximates TG(ω) as training proceeds. In the early stage of training, as shown in (**b**), the approximation error is large, but the updated parameters have a wide range; in the middle and later stages of training, as shown in (**c**,**d**), the approximation error gradually decreases, and the updated parameters are gradually concentrated around zero.

The derivative of TG(ω) at any point except for the ω=0 is 0, so the function is not suitable for the gradient optimization process. It is necessary to approximate the gradient of TG(ω) so that it can be used for gradient descent. Kim et al. [46] directly use 1 to approximate the gradient of TG(ω), which called identity approximation. It can be observed from Figure 3a that such approximation is very rough, which brings a large error due to the gradient mismatch. We introduce an asymptotic approximation function, denoted A(ω), which is inspired by the Error Decay Estimator (EDE) method proposed in [47], namely,

(3) TG(ω)≈A(ω)=k·(tanh(t·ω)+1)

where $t=T_{\min}10^{\frac{i}{N}\times \log\left(\frac{T\max}{T\min}\right)}$, $k=\frac{1}{2}\max\left(\frac{1}{t},1\right)$, and $T_{\min}=0.1,T_{\max}=10$. In addition, *N* denotes all the epochs required for training, *i* represents the current epoch, *k* and *t* control the variation of A(ω) during the training process. Thus, the gradient of TG(ω) can be approximated as

(4) $$\frac{\partial TG(\omega )}{\partial \omega }\approx \frac{\partial A(\omega )}{\partial \omega }=k\cdot t\cdot \left(1-\tanh{\left(t\cdot w\right)}^{2}\right)$$

In conclusion, we construct a gate function that can be trained using gradient descent. By approximating the gate function TG(ω) asymptotically, the error can be reduced without sacrificing the ability of updating parameters.

#### 3.3.2. Continuous Approximation for Discrete One-Hot Vector

In this section, a continuous one-hot mask vector $OH_{\alpha^{l}}$ (*l* is the layer index) is constructed based on TG(ω). The convolutional operator for each branch is defined as $OP_{i}$ and the length of $OH_{\alpha^{l}}$ is equal to the number of branches. To construct $OH_{\alpha^{l}}$, additional $\left\lceil log_{2}N\right\rceil$ parameters (⌈·⌉ means upward rounding) need to be introduced, which are named architecture parameters $\alpha^{l}$ and expressed as $\left[\alpha_{0}^{l},\alpha_{1}^{l},\ldots ,\alpha_{\lceil log_{2}N\rceil -1}^{l}\right]$. Then, $OH_{\alpha^{l}}$ can be constructed using Equations (5) and (6), where $OH_{\alpha^{l}}\left(i\right)$ represents the *i*th element of $OH_{\alpha^{l}}$.

(5) $$OH_{\alpha^{l}}\left(i\right)=\prod_{j=0}^{\lceil \log_{2}N\rceil -1}\left(B_{j}\cdot TG\left(\alpha_{j}^{l}\right)+\left(1-B_{j}\right)\cdot \left(1-TG\left(\alpha_{j}^{l}\right)\right)\right)$$

(6) $$B_{j}=\left\{\begin{array}{cc} \;\left\lfloor \frac{i}{2^{\lceil \log_{2}N\rceil -1}}\right\rfloor , & j=0\\ \left\lfloor \frac{i\%2^{\left\lceil \mathrm{lo}g_{2}N\right\rceil -1}\%\ldots \%2^{\left\lceil \mathrm{lo}g_{2}N\right\rceil -j}}{2^{\left\lceil \mathrm{lo}g_{2}N\right\rceil -1-j}}\right\rfloor & ,1\leq j\leq \left\lceil \mathrm{lo}g_{2}N\right\rceil -1 \end{array}\right.$$

In the above equation, ⌊·⌋, % and ∏ represent downward rounding, remainder operation and multiplication operation, respectively. Next, the gradient of the architecture parameters $\alpha^{l}$ will be analyzed. Supposing the input tensor of the *l*th layer is $x^{l}$ and the output tensor is $y^{l}$, which can be expressed as $y^{l}=OS^{l}\left(x^{l}\right)=\sum_{i=0}^{N-1}OH_{\alpha^{l}}\left(i\right)\cdot OP_{i}\left(x^{l}\right)$

Then, the gradient of $\alpha_{j}^{l}$ is

(7) $$\frac{\partial y^{l}}{\partial \alpha_{j}^{l}}=\sum_{i=0}^{N-1}\frac{\partial OH_{\alpha^{l}}\left(i\right)}{\partial \alpha_{j}^{l}}\cdot OP_{i}\left(x^{l}\right),j\in \left\{0,1,\ldots ,\left\lceil \log_{2}N\right\rceil -1\right\}$$

where

$$\frac{\partial OH_{\alpha^{l}}\left(i\right)}{\partial \alpha_{j}^{l}}=\left(2B_{j}-1\right)\cdot \frac{\partial TG\left(\alpha_{j}^{l}\right)}{\partial \alpha_{j}^{l}}\prod_{g=0,g\neq j}^{\left\lceil \log_{2}N\right\rceil -1}\left(B_{g}\cdot TG\left(\alpha_{g}^{l}\right)+\left(1-B_{g}\right)\cdot \left(1-TG\left(\alpha_{g}^{l}\right)\right)\right).$$

In the backpropagation process, $\alpha^{l}$ can be updated using the gradient obtained above. In conclusion, we construct a continuous one-hot mask vector $OH_{\alpha^{l}}$ using the architecture parameters $\alpha^{l}$ and derive the gradient of $\alpha^{l}$ to update $\alpha^{l}$ and $OH_{\alpha^{l}}$.

#### 3.3.3. Resource-Constrained Objective Function

The objective function is described as

(8) $$\arg\min_{\theta ,\alpha }L\left(\theta ,\alpha \right)+\lambda \cdot R\left(\alpha \right)$$

θ and α indicate the parameters of convolutional operators and architecture parameters, respectively. L(θ,α) is the cross-entropy loss function used to measure the classification error of the model during the training process. R(α) is a regularization term used to measure the number of model parameters and FLOPs, which is only related to α. λ is the corresponding regularization factor.

In the following, we compute the regularization term R(α) for the reconstructed CNN. For the ith operator $OP_{i}$ in the *l*th layer, the number of parameters and FLOPs can be represented as $PS_{i}^{l}$ and $FP_{i}^{l}$. Detailed equations are shown in Appendix A. Then, the total number of parameters and FLOPs of lth layer can be expressed as $PS^{l}=\sum_{i=0}^{N-1}OH_{\alpha^{l}}\left(i\right)\cdot PS_{i}^{l}$ and $FP^{l}=\sum_{i=0}^{N-1}OH_{\alpha^{l}}\left(i\right)\cdot FP_{i}^{l}$, respectively. So far, the corresponding regularization term $R^{l}\left(\alpha^{l}\right)$ of the lth layer can be denoted as $R^{l}\left(\alpha^{l}\right)=\frac{1}{2}\left(\log\left(PS^{l}\right)+\log\left(FP^{l}\right)\right)$. The total regularization term R(α) is equal to the sum of $R^{l}\left(\alpha^{l}\right)$ over all layers, and L is the number of CNN layers, we can obtain $R\left(\alpha \right)=\sum_{l=1}^{L}R^{l}\left(\alpha^{l}\right)$.

### 3.4. The Fine-Tuning Stage

**Figure 4:**
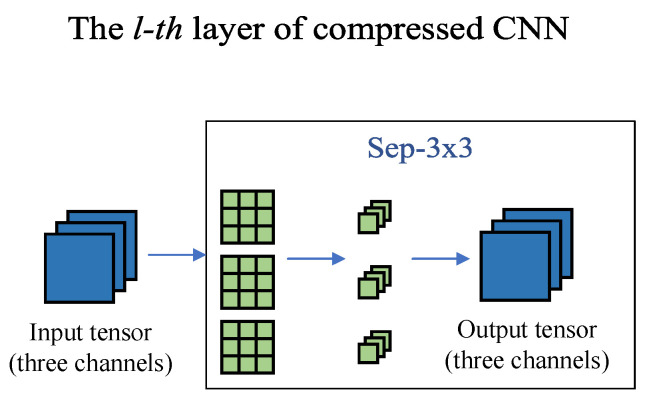
Illustration of the fine-tuning stage. Leaving only the branch with a mask of 1 in each layer and fine-tuning the compressed CNN for several epochs.

When the training in the searching stage is completed, all the branches with masks of 0 in the reconstructed CNN will be removed, only leaving the branch with a mask of 1 in each layer, as shown in Figure 4. Such behavior does not degrade the model performance, because those branches do not work. However, due to the potential problem of inadequate training in the searching stage, we perform the fine-tuning process on the pruned CNN to further improve performance. After the fine-tuning stage, the lightweight CNN is obtained.

## 4. Experiments

### 4.1. Dataset

Cifar-10 [48]: The dataset has 60,000 images, each of which is an RGB three-channel image with a size of 32 × 32. It has 10 categories, with 6000 images per category. The dataset is divided into a training set and a validation set containing 50,000 and 10,000 images, respectively.

Cifar-100 [48]: The dataset also has 60,000 images, each of which is an RGB three-channel image with a size of 32 × 32. It has 100 categories with 600 images per category. The dataset is also divided into a training set and a validation set containing 50,000 and 10,000 images, respectively.

ImageNet-160-120 [49,50]: The dataset is built from ImageNet 16 × 16 [49], which is a down-sampled variant of ImageNet. The spatial resolution of ImageNet 16 × 16 is 16 × 16, and ImageNet-160-120 is constructed by selecting all images with label ∈[1,120] from ImageNet 16 × 16. Chrabaszcz [49] has proved that down-sampling images in ImageNet can significantly reduce computation costs for solving the optimal hyper-parameters of some classical models while maintaining similar search results. In summary, ImageNet-160-120 contains 151.7 K training images and 6 K testing images with 120 classes.

### 4.2. Evaluation Metrics

We use the compression rate and TOP1 accuracy as evaluation metrics. The parameters compression ratio (PCR) and FLOPs compression ratio (FCR) are used to measure compression degree of a CNN model. PCR indicates the ratio of the number of parameters in the original CNN to the number of parameters in the compressed one and FCR indicates the ratio of the FLOPs in the original CNN to the FLOPs in the compressed one. The larger the PCR is, the smaller the model size will be. The larger the FCR is, the faster the model can be computed.

The formula for PCR and FCR are:

(9) $$PCR=\frac{Params_{original}}{Params_{compressed}}$$

(10) $$FCR=\frac{{FLOPs}_{original}}{{FLOPs}_{compressed}}$$

### 4.3. Results on Cifar

We compress ResNet20, ResNet56, VGG16 on Cifar-10 and ResNet18 on Cifar-100. For those CNNs, we do not compress the first convolutional layer and the last fully connected layer. The compression metrics, such as PCR and FCR, are calculated over all layers except the first convolutional layer and the last fully connected layer. The number of channels per convolutional layer for those CNNs is shown in Table 1.

The parameters θ of convolutional operators are optimized using the SGD optimizer with momentum, where the initial learning rate is 0.1, the momentum is 0.9 and the weight decay is $3\times 10^{-4}$. The learning rate is set using the CosineAnnealingLR scheme in Pytorch [51]. The architecture parameters α are optimized using Adam optimizer with an initial learning rate of 0.01 and a weight decay of $10^{-3}$, with the learning rate decaying by a factor of 0.3 every 40 epochs. Batch size is 256, and the total training epochs are 150. The architecture parameter α was randomly initialized using a normal distribution with mean 0 and variance 0.01. The parameters θ and α are jointly trained.

**Figure 5:**
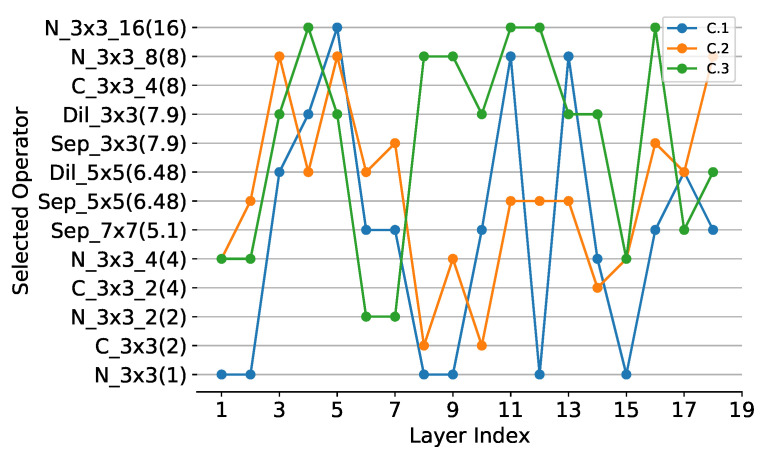
The operator of each layer in compressed ResNet20. The layers in the figure do not include the first convolutional layer and the last fully connected layer. From bottom to top on the vertical axis, the operators are increasingly lightweight. The float number in (·) represents the lightness of operator, the higher the value is, the lighter the operator is. The model located in the upper part of the figure is more lightweight.

**Figure 6:**
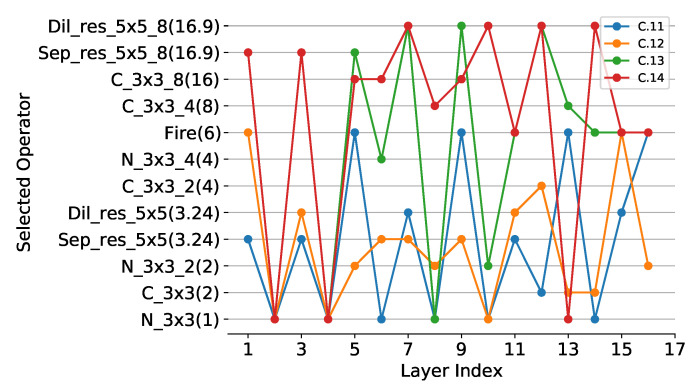
The operator of each layer in compressed ResNet18. The layers in the figure do not include the first convolutional layer and the last fully connected layer. The vertical axis has the same meanings as Figure 5.

**Figure 7:**
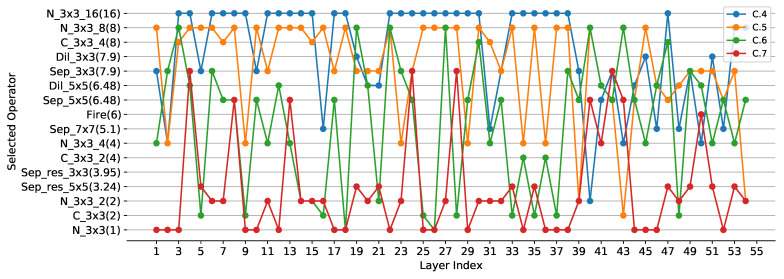
The operator of each layer in compressed ResNet56. The layers in the figure do not include the first convolutional layer and the last fully connected layer. The vertical axis has the same meanings as Figure 5.

**ResNet20 on Cifar-10:** In ResNet20, convolutional operators with strides 1 and 2 are reconstructed to selection operators with strides 1 and 2, respectively. Different SOPs are used in compression experiments, and detailed information about SOPs is shown in Appendix C. ResNet20 is already a compact CNN, so there are few compression experiments on it. Therefore, we compare it directly with the baseline results. We use SOP1 and SOP2 to perform compression experiments on ResNet20, and set λ to 0 and $1.5^{-3}$ respectively. Simple operators are used in SOP1 and SOP2, and the meanings of the operators can be found in Appendix A. SOP2 is more lightweight than SOP1. In the case of the same λ, it can be seen that the lighter the SOP is, the higher the compression degree of CNN will be, but the more the accuracy will drop. When using the same set of operators, the larger the λ is, the higher the compression degree and accuracy drop will be, as shown in Table 2. It is consistent with common sense. The MA column in Table 2 represents the architecture of the compressed lightweight CNN, which can be found in Figure 5, Figure 6 and Figure 7.

**ResNet56 on Cifar-10:** The reconstruction of over-parameterized CNN for ResNet56 is similar to ResNet20. We use SOP1, SOP2 and SOP3 to perform compression experiments on ResNet56, and set λ to 0 and $1.5^{-3}$, respectively. Complex operators have been added to SOP3, including the Fire module, the Sep_res_3×3 module and the Sep_res_5×5 module. The meanings of these operators can be found in Appendix A. When using SOP1 with λ set to $1.5^{-3}$, PCR and FCR of compressed model are 5.25 and 5.96, respectively, with TOP1 accuracy being 91.96, as shown in Table 2. Keeping λ constant, PCR and FCR increase to 6.59 and 7.94, respectively, when using a lighter SOP2, while TOP1 accuracy drops to 91.22. When using SOP1 with λ reduced to 0, PCR and FCR decrease to 4.4 and 3.37 and TOP1 accuracy rises to 92.5, respectively. It can be seen that the larger the λ is, the higher the compression degree of the model will be, but the more the accuracy will drop. When using SOP3 with complex operators, model accuracy of 93.75 can be achieved, indicating that the SOP is critical to the compression. For two operators of similar lightness, the complex operator is superior to the single operator.

**VGG16 on Cifar-10:** The convolutional operator in VGG16 is reconstructed to the selection operator with stride 1 and the pooling operator is reconstructed to the selection operator with stride 2. We use SOP4, SOP5 and SOP6 to perform compression experiments on VGG16. The meanings of the complex operators added in SOP4 and SOP5 can be found in Appendix A. SOP5 sets the group number of the group convolution in the complex operator to 1, which has more parameters relative to the complex operator in SOP4. When using SOP5 with λ set to 0, the compressed model can achieve PCR and FCR of 2.82 and 3.71, respectively, with TOP1 accuracy being 94.65. To further improve the degree of compression, the 1×1 convolutional operator in the complex operators such as Sep_res_3×3, Sep_res_5×5, Dil_res_3×3 and Dil_res_5×5 is modified to a group convolution operator with the group number of 4 to construct SOP4. As a result that these complex operators consist of depthwise separable convolution and 1 × 1 convolution operators, the 1 × 1 convolution operator plays a dominant role in the number of parameters and FLOPs in these complex operators when the number of convolution channels is large. When SOP4 is used to compress VGG16, PCR and FCR increase to 3.85 and 2.61, respectively, while TOP1 accuracy decreases to 93.95. PCR and FCR can even increase to 15.1 and 15.6 when using lighter SOP6, however, TOP1 accuracy drops to 92.35.

**ResNet18 on Cifar-100:** The reconstruction of over-parameterized CNN for ResNet18 is similar to ResNet20. We use SOP7 and SOP8 to perform compression experiments on ResNet18, and set λ to 0 and $1.5^{-3}$, respectively. Compared to SOP7, SOP8 uses two more lightweight operators C_3×3_4 and C_3×3_8. The compressed model can achieve PCR and FCR of 2.39 and 2.23 by using SOP7 with λ set to 0 and the TOP1 accuracy is 74.3, the obtained model architecture is shown in Figure 6. C.11. When increasing λ to $1.5^{-3}$, PCR, FCR and TOP1 accuracy are 2.44, 2.34 and 74.2. To further improve the degree of compression, we use SOP8 and set λ to 0. The PCR and FCR of the compressed model are 5.27 and 2.97, and surprisingly the TOP1 accuracy is 74.85. When λ is set to $1.5^{-3}$, PCR, FCR and TOP1 accuracy are 4.66, 3.98 and 73.6, as shown in Table 2.

### 4.4. Results on ImageNet

We compress DenseNet121 [54], MobileNetV2 [3] on ImageNet-16-120. Since the spatial resolution of ImageNet16×16 is 16×16, we reserve only one downsampling layer with a stride 2 in these two models. In addition, we revise the classification layer from 1000D fully-connected to 120D fully-connected. We present their architectures in Table 3 and Table 4, respectively. For DenseNet121, we only compress 3×3 convolutional operators in Dense Block. For MobileNetV2, we only compress those 1×1 convolutional operators in bottleneck. We do not compress the first convolutional layer and the last fully connected layer too. The experimental hyperparameters are the same with the experiments on Cifar, except that the batch size is modified to 512.

**DenseNet121 on ImageNet-16-120:** In DenseNet121, those 3×3 convolutional operators in Dense Block are reconstructed to selection operators with stride 1. We select the set of lightweight operators SOP9 for Densenet121 and then compress the model using different λ. The model size can be compressed by 3.42 times with a 0.23% decrease in accuracy when λ is set to 0. It is surprising that as λ increases to $1.5\times 10^{-4}$, not only does the compression rate increase to 5.13, but the accuracy also increases by 0.13%. As λ keeps growing, the compression rate will continue to rise, along with more serious performance degradation. Furthermore, if λ is smaller than $1.5\times 10^{-3}$, a significant reduction in model size can be obtained by raising λ. However, once λ exceeds $1.5\times 10^{-3}$, the compression effect gained by increasing λ will no longer be remarkable. More results are shown in Table 5.

**MobileNetV2 on ImageNet-16-120:** In MobileNetV2, we only compress those 1×1 convolutional operators in bottleneck, as the contribution of 3×3 depthwise separable convolution to model size is negligible. The lightweight operator set we used is SOP10, which consists of 1×1 group convolution, Fire, 3×3 group convolution and 3×3 CReLU group convolution. The detailed operators are given in the Appendix C. It is worth to notice that all the operators except the 1×1 group convolution in SOP10 have more parameters than the original N_1×1 convolution. For instance, the parameter volumes of Fire and N_3×3_8 are 1.5 and 1.12 times larger than N_1×1, respectively, with the same channel dimension. Hence, the model can achieve being compressed only when λ is sufficiently large. As shown in Table 5, when λ is 0 and $1.5\times 10^{-4}$, although the accuracy of the obtained model is improved, the size is also larger than the original one. The model compression rate increases to 1.48 when λ increases to $5\times 10^{-4}$, and the accuracy also improves to 49.87. Similarly, with increasing λ, there will be worse performance, although the model compression rate will continue to increase. As λ proceeds to rise beyond $5\times 10^{-3}$, the additional compression gain will be negligible.

### 4.5. Ablation Study

**Figure 8:**
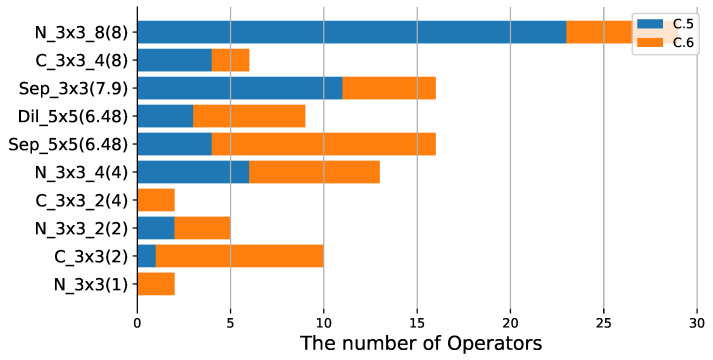
The distribution of operators in SOP1 when compressing resnet56 on Cifar10. C.5 and C.6 correspond to λ of $1.5^{-3}$ and 0, respectively. N_3×3_8 appears the most times in this experiment, indicating that N_3×3_8 has a better effectiveness. As λ increases, the algorithm tends to select the more lightweight operators, with blue histograms located in the upper part of the figure.

**Figure 9:**
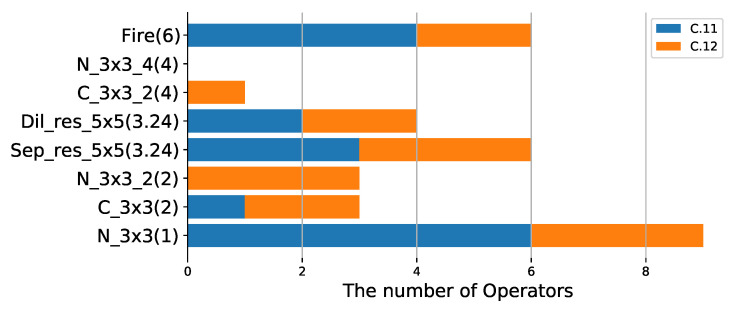
The distribution of operators in SOP7 when compressing resnet18 on Cifar100. C.11 and C.12 correspond to λ of 0 and $1.5^{-3}$, respectively. Compared to simple operators, complex operators are more likely to be selected in compression training, indicating that complex operators are more effective, such as Fire, Sep_res_5×5 and Dil_res_5×5.

**Operation selection analysis:** To evaluate the effectiveness of different lightweight operators in our method, experiments using different SOPs are performed without changing λ, and the results are shown in Table 2 and Table 5. We can find that using the lighter SOP yields a more compact model, for example, C.6 with SOP1 located above C.7 with SOP3 in Figure 7 since SOP1 is lighter than SOP3. The same conclusion can be derived from Figure 5 and Figure 6. In addition, to study the performance of different operators, we perform multiple experiments with the same SOPs and different λ. It can be visualized from Figure 8 that N_3×3_8 is used most frequently in the compressed model, indicating that it is more effective, followed by Sep_3×3 and Sep_5×5. In addition, $N\_3\times 3\_g_{1}$ has the same lightness compared with $C\_3\times 3\_g_{2}$ ($g_{1}=2g_{2}$), however, $N\_3\times 3\_g_{1}$ is more likely to be selected during training, suggesting that $N\_3\times 3\_g_{1}$ is more effective. Compared with simple operators, complex operators are more effective, such as Fire [1], Sep_res_5×5 and Dil_res_5×5, as shown in Figure 9.

From Figure 6 to Figure 7, it is interesting to see that the operators with higher lightness (e.g., N_3×3_16) tend to appear in the shallower layers (close to the input of models), while the operators with lower lightness (e.g., N_3×3) tend to appear in the deeper layers (close to the output of models). In our opinion, it is mainly due to the fact that shallow feature maps have higher resolution which leads to more redundant information, while deep feature maps have lower resolution and thus less redundant information. In addition, there is an alternation between operators of different lightness. The operators of higher lightness are often followed by several operators of lower lightness. We assume that appropriate redundancy is useful for training convergence. If the operators are too lightweight to extract sufficient information, it will tend to follow the less lightweight operators to regain more information, thus compensating for the model performance.

**Figure 10:**
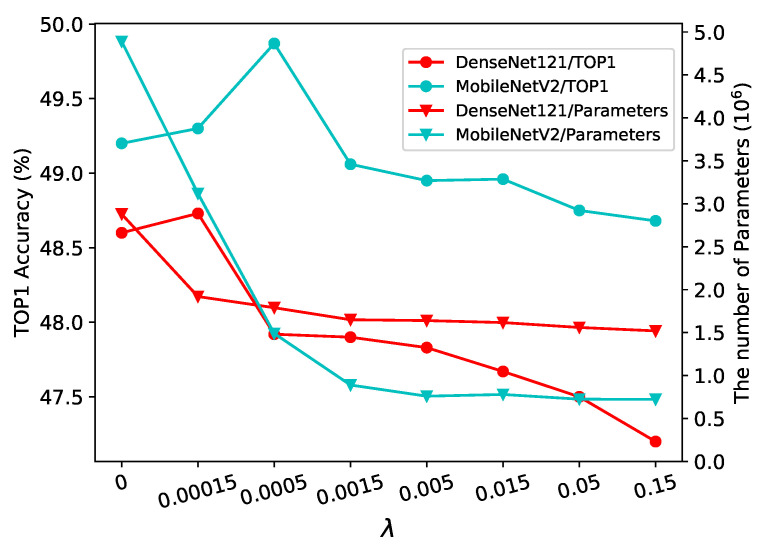
The effect of λ on model size and TOP1 accuracy. For ease of visualization, the λ-axis is displayed with a uniform scale.

**Effect of λ:** The compression rate is influenced by both SOP and λ. Once SOP has been selected, the maximum achievable compression rate is determined. As can be seen from Figure 10, the compression rate gradually grows as λ increases, however, once λ reaches a certain threshold, the improvement of compression rate will be insignificant. In this case, the compression rate is close to the maximum achievable value and the only choice to further raise the compression rate is to select a lighter SOP. Moreover, the threshold is related to the SOP, the lighter the SOP is, the smaller the threshold will be. From Figure 10, it is evident that the threshold is $1.5\times 10^{-3}$ for Densenet121 and $5\times 10^{-3}$ for MobileNetV2 since SOP9 is more lightweight than SOP10. λ can prevent over-fitting, which is similar to what dropout does. It is clear that when λ is relatively small, not only does the compression rate rise as λ increases, but the accuracy of the model is also elevated. After λ arrives at a critical value, the compression rate will continue to grow as λ keeps increasing, but the model accuracy will begin to drop. Likewise, the critical value is also related to SOP, the lighter the SOP is, the smaller the critical value will be. The critical value of λ is $1.5\times 10^{-4}$ for Densenet121 and $5\times 10^{-4}$ for MobileNetV2.

**Algorithm extensibility:** Our proposed differentiable selection operator is similar to common convolutional operator that can be trained using gradient descent directly. It not only can be embedded in a classification CNN, but also in a detection or segmentation CNN. Thus, our proposed compression algorithm is task-independent, and it not only can be applied to classification tasks but also to other vision tasks such as detection and segmentation. Furthermore, besides CNN, our proposed differentiable selection operator can be embedded in any networks that can be optimized by gradient descent, such as recurrent neural networks (RNN), generative adversarial networks (GAN), etc. From an intrinsic perspective, we propose a continuous approximation method for discrete one-hot vectors, which can be used not only for the model compression presented in this paper, but also for the network architecture search (NAS). In addition, the method can also be used for model quantization. If we use operators with different quantization bit widths to construct the selection operator, then we can obtain a mixed-precision quantization model after training.

**Algorithm complexity:** Selecting suitable operators manually to form a differentiable selection operator is the key point of our proposed compression algorithm. Fortunately, many lightweight operators are available for us. Once the selection operator is constructed, we can directly replace the original operator in the network to be compressed with it to construct an over-parameterized network. Then, we can train the network as we train a normal one. In addition, once the lightweight operators are selected, we only need to modulate λ to achieve different levels of compression without setting the compression rate separately for each layer. Therefore, the algorithm is easy to implement. Compared to the original network, the over-parameterized network takes more time and memory to train, since each selection operator in it has N branches. Fortunately, we can alleviate the aforementioned problem. From the perspective of forward propagation, the output of selection operator is $y=OS\left(x\right)=\sum_{i=0}^{N-1}OH_{\alpha }\left(i\right)\cdot OP_{i}\left(x\right)$. Since the mask vector $OH_{\alpha }$ is a one-hot vector, **y** is equal to the output of the branch $OP_{i}$ corresponding to $OH_{\alpha }\left(i\right)=1$, irrelevant to all other branches. From the perspective of back propagation, the update of architecture parameter $\alpha_{j}$ is only related to those branches with $\frac{\partial OH_{\alpha }\left(i\right)}{\partial \alpha_{j}}\neq 0$ according to Equation (7). Thus, we only need to compute those branches with $OH_{\alpha }\left(i\right)=1$ or $\frac{\partial OH_{\alpha }\left(i\right)}{\partial \alpha }\neq 0$ at each step. When there are 8 branches, only 4 branches are calculated on average at each step. This reduces memory consumption and training time significantly. For smaller models on Cifar, the compression process usually takes only 3–4 GPU hours, while for larger models on ImageNet, it usually takes 8–10 GPU hours.

## 5. Conclusions

A differentiable algorithm is proposed in this paper for CNN compression. Different from previous methods that prune redundant units from bulky convolutional operators, our method addresses the CNN compression problem from a completely new perspective by directly replacing the original bulky convolutional operators with more lightweight ones. The proposed approach is an end-to-end compression method that only requires control λ to achieve different levels of compression, without specifying the compression rate for each layer. Specifically, our method can break the constraints of fixed operators in the network and obtain a higher compression rate without significant performance degradation. For example, the compressed ResNet20 and ResNet56 retain only 0.11 M and 0.24 M parameters, respectively, but their performance still outperforms the original network. A thorough comparison with several state-of-the-art compression methods proved the superiority of our proposed methodology on several highly competitive datasets. Overall, the proposed approach shows a unique potential for using gradient descent to seek the best lightweight operator for each layer to achieve compression, thus facilitating the application of CNNs on mobile and embedded devices.

## Figures and Tables

**Table 1:** The number of channels in ResNet20, ResNet56, VGG16 and ResNet18.

ResNet20				
Conv	conv1-6	conv7-12		conv13-18
Channel	16	32		64
ResNet56				
Conv	conv1-18	conv19-36		conv37-54
Channel	16	32		64
VGG16				
Conv	conv1-2	conv3-4	conv5-7	conv8-13
Channel	64	128	256	512
ResNet18				
Conv	conv1-4	conv5-8	conv9-12	conv13-16
Channel	64	128	256	512

**Table 2:** Results of compressing ResNet20, ResNet56, VGG16 on Cifar-10 and ResNet18 on Cifar-100. SOP represents the set of operators used in the reconstructed CNN. TOP1 refers to the TOP1 accuracy of the model on the test set. MA is the architecture of the compressed model, as shown in Figure 5, Figure 6 and Figure 7. Paras represents the number of parameters in the CNN, containing the number of parameters of all convolutional layers except the first convolutional layer and the last fully connected layer.

Model	Method	SOP	λ	PCR	FCR	Paras	TOP1 (%)	MA
ResNet20	He. [45]	-	-	0	0	0.27 M	91.25	-
	Ours	SOP2	0	2.87	2.56	0.11 M	91.6	C.1
		SOP1	$1.5\times 10^{-3}$	4.91	4.19	0.06 M	90.35	C.2
		SOP2	$1.5\times 10^{-3}$	6.48	5.61	0.047 M	90.15	C.3
ResNet56	He. [45]	-	-	0	0	0.85 M	93.03	-
	Li. [18]	-	-	1.16	1.38	-	93.06	-
	Dug. [52]	-	-	-	2.12	-	92.72	-
	Ours	SOP3	0	3.51	3.09	0.24 M	93.75	C.7
		SOP1	0	4.4	3.37	0.19 M	92.5	C.6
		SOP1	$1.5\times 10^{-3}$	5.25	5.96	0.17 M	91.96	C.5
		SOP2	$1.5\times 10^{-3}$	6.59	7.94	0.14 M	91.22	C.4
VGG16	Simon. [53]	-	-	0	0	16.3 M	93.25	-
	Li [18]	-	-	2.78	1.52	-	93.4	-
	Dug. [52]	-	-	17.12	3.15	-	92.85	-
	Ours	SOP5	0	2.82	3.71	5.79 M	94.65	C.8
		SOP4	0	3.85	2.61	4.23 M	93.95	C.9
		SOP6	$1.5\times 10^{-3}$	15.1	15.6	1.08 M	92.35	C.10
ResNet18	He. [45]		-	0	0	11 M	75.05	-
	Ours	SOP7	0	2.39	2.23	4.61 M	74.5	C.11
		SOP7	$1.5\times 10^{-3}$	2.44	2.31	4.5 M	74.2	C.12
		SOP8	0	5.27	2.97	2.08 M	**74.85**	C.13
		SOP8	$1.5\times 10^{-3}$	4.66	3.98	2.36 M	73.6	C.14

**Table 3:** DenseNet121 architectures for ImageNet-16-120.

Layers	Output Size	Stride	Densenet121
conv	16 × 16	1	3 × 3 conv
Dense Block (1)	16 × 16	1	[3 × 3 conv] × 6
Transition Layer (1)	16 × 16	1	1 × 1 conv 2 × 2 average pool
Dense Block (2)	16 × 16	1	[3 × 3 conv] × 12
Transition Layer (2)	16 × 16	1	1 × 1 conv 2 × 2 average pool
Dense Block (3)	16 × 16	1	[3 × 3 conv] × 24
Transition Layer (3)	8 × 8	2	1 × 1 conv 2 × 2 average pool
Dense Block (4)	8 × 8	1	[3 × 3 conv] × 16
Classification Layer	1 × 1	-	8 × 8 average pool 120D fully-connected

**Table 4:** MobileNetV2 architectures for ImageNet-16-120. In the table, bottleneck is $\left[1\times 1\mathrm{conv},3\times 3{\;}_{-}\mathrm{conv},1\times 1\right.$ conv ], where $3\times 3\;{\mathrm{dw}}_{-}\mathrm{conv}$ indicates 3×3 depthwise separable convolution. The meaning of Expansion Ratio can be seen from [3].

Layers	Output Size	Stride	Channels	Expansion Ratio	MobilenetV2
conv	16 × 16	1	32	-	3 × 3 conv
Block (1)	16 × 16	1	16	1	bottleneck × 1
Block (2)	16 × 16	1	24	6	bottleneck × 2
Block (3)	16 × 16	1	32	6	bottleneck × 3
Block (4)	16 × 16	1	64	6	bottleneck × 4
Block (5)	16 × 16	1	96	6	bottleneck × 3
Block (6)	8 × 8	2	160	6	bottleneck × 3
Block (7)	8 × 8	1	320	6	bottleneck × 1
conv	8 × 8	1	1280	-	1 × 1 conv
Classification Layer	1 × 1	-	-	-	8 × 8 average pool 120Dfully-connected

**Table 5:** Results of compressing DenseNet121 and MobileNetV2 on ImageNet-16-120.

Model	Method	SOP	λ	PCR	Paras	TOP1 (%)	MA
DenseNet121	Huang. [54]	-	-	0	9.84 M	48.83	-
	Ours	SOP9	0	3.42	2.88 M	48.6	C.15
		SOP9	$1.5\times 10^{-4}$	5.13	1.92 M	48.73	C.16
		SOP9	$5\times 10^{-4}$	5.50	1.79 M	47.92	C.17
		SOP9	$1.5\times 10^{-3}$	5.96	1.65 M	47.9	C.18
		SOP9	$5\times 10^{-3}$	6.0	1.64 M	47.83	C.19
		SOP9	$1.5\times 10^{-2}$	6.09	1.617 M	47.67	C.20
		SOP9	$5\times 10^{-2}$	6.31	1.56 M	47.5	C.21
		SOP9	$1.5\times 10^{-1}$	6.47	1.52 M	47.2	C.22
MobileNetV2	sandler. [3]	-	-	0	2.21 M	49.2	-
	Ours	SOP10	0	0.45	4.89 M	49.3	C.23
		SOP10	$1.5\times 10^{-4}$	0.71	3.12 M	49.4	C.24
		SOP10	$5\times 10^{-4}$	1.48	1.49 M	49.87	C.25
		SOP10	$1.5\times 10^{-3}$	2.48	0.89 M	49.06	C.26
		SOP10	$5\times 10^{-3}$	2.91	0.76 M	48.95	C.27
		SOP10	$1.5\times 10^{-2}$	2.83	0.78 M	48.96	C.28
		SOP10	$5\times 10^{-2}$	3.05	0.725 M	48.75	C.29
		SOP10	$1.5\times 10^{-1}$	3.06	0.723 M	48.68	C.30

**Figure A1:**
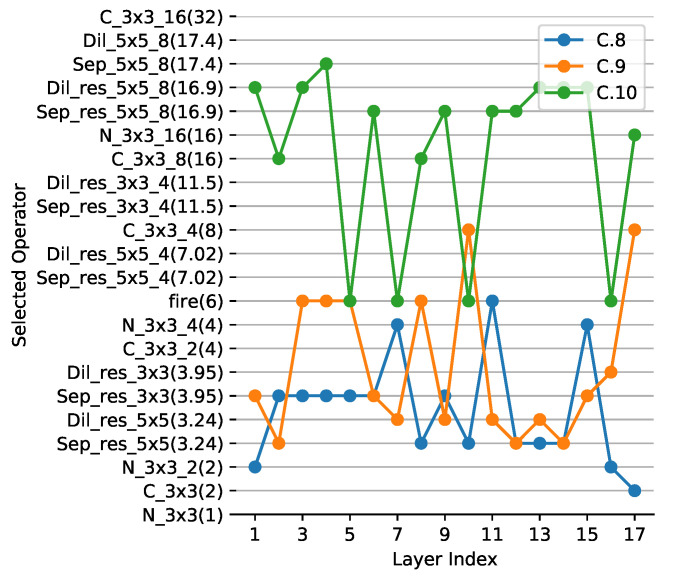
The operator of each layer in compressed VGG16. The layers in the figure do not include the first convolutional layer and the last fully connected layer. The vertical axis have the same meanings as Figure 5.

**Figure A2:**
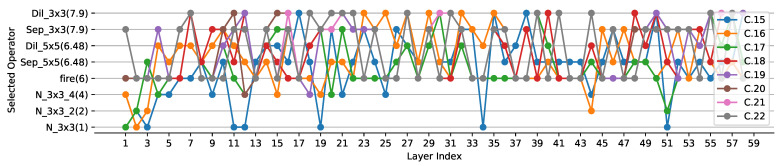
The operator of each layer in compressed DenseNet121. The layers in the figure do not include the first convolutional layer and the last fully connected layer. The vertical axis has the same meanings as Figure 5.

**Figure A3:**
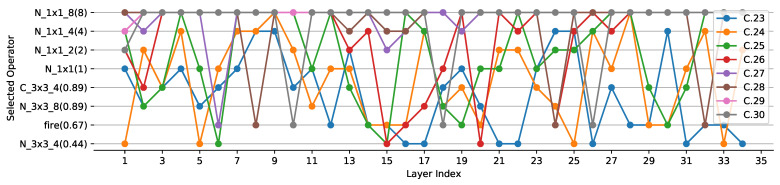
The operator of each layer in compressed MobileMetV2. The layers in the figure do not include the first convolutional layer and the last fully connected layer. The vertical axis has the same meanings as Figure 5.

## Data Availability

Not applicable.

## Appendix A. Operators

We experiment with different operators, and Table A1 lists the specific settings of the different operators and the corresponding number of parameters and FLOPs.

**The description of simple symbols:**

**$C_{i}$**: Number of input channels; $C_{o}$: Number of output channels; S: stride; P: padding; G: Number of groups; d: dilation; A: activation function; K: kernel size; PS: Number of parameters; Flops: floating-point operations; H,W: The height and width of the input tensor.

**N_3 × 3_g** denotes a normal convolutional operation, where the number of groups g can be 1, 2, 4, 8, 16, 32, …, and the group convolution is followed by the channel shuffle operation;

**N_1 × 1_g** is similar to N_3 × 3_g, except that the kernel size is 1;

**skip_connect** denotes a shortcut that directly connects two nodes with the same input and output;

**avg_pool_3× 3** denotes average pooling;

**max_pool_3 × 3** denotes maximum pooling;

**Sep_k × k** denotes a depthwise separable convolution consisting of two convolutional layers, the first being a depthwise separable convolution with a convolutional kernel of k × k and the second being a 1 × 1 convolution;

**Dil_k × k**: denotes a dilated convolution consisting of two convolutional layers, the first of which is a depthwise separable dilated convolution with a convolutional kernel of k × k and the second of which is a 1 × 1 convolution;

**C_3 × 3_g** is similar to N_3 × 3_g, except that the activation function is replaced by CReLU from ReLU, where the number of groups can be 1,2,4,8, 16, 32, ..., the group convolution is followed by the channel shuffle operation;

**The description of complex symbols:**

**Fire**: Using the Fire module in SqueezeNet, which consists of a squeeze layer with a convolutional kernel of 1×1 and two expand layers with a convolutional kernel of 1×1 and 3 × 3, respectively, where the number of input channels in the squeeze layer is $C_{in}$ and the number of output channels is $C_{squeeze}$; the number of input channels in the expand layer is $C_{squeeze}$. The number of output channels is $\frac{C_{out}}{2}$, and the output of the Fire concatenates the outputs of the two expand layers together before outputting them. Here, we define $C_{squeeze}$ as $min(\frac{C_{out}}{4},64)$;

**Sep_res_k × k_g**: Connect the two Sep_k × k convolutional operators using residuals, and that the 1×1 convolution layer in Sep_k × k uses group convolution with a group number of g, followed by the operation of channel shuffle;

**Dil_res_k × k_g**: Connect the two Dil_k × k convolutional operators using residuals, and that the 1×1 convolution layer in Dil_k × k uses group convolution with a group number of g, followed by the operation of channel shuffle.

**Table A1:** Different operators used in our experiments.

Name	$C_{i}$	$C_{o}$	S	P	G	d	A	k	PS	Flops
N_3 × 3_g	$C_{i}$	$C_{o}$	S	1	g	1	ReLU	3 × 3	$\frac{9\cdot C_{i}\cdot C_{o}}{g}$	PS·H·W
N_1 × 1_g	$C_{i}$	$C_{o}$	S	1	g	1	ReLU	1 × 1	$\frac{C_{i}\cdot C_{o}}{g}$	PS·H·W
skip_connect	$C_{i}$	$C_{i}$	-	-	-	-	-	-	0	0
avg_pool_3 × 3	$C_{i}$	$C_{i}$	S	1	1	1	ReLU	3 × 3	0	$9\cdot C_{i}\cdot H\cdot W$
max_pool_3 × 3	$C_{i}$	$C_{i}$	S	1	1	1	ReLU	3 × 3	0	$9\cdot C_{i}\cdot H\cdot W$
Sep_3 × 3	$C_{i}$	$C_{o}$	S	1	$C_{i}$	1	ReLU	3 × 3	$9\cdot C_{i}+C_{i}\cdot C_{o}$	PS·H·W
Sep_5 × 5	$C_{i}$	$C_{o}$	S	2	$C_{i}$	1	ReLU	5 × 5	$25\cdot C_{i}+C_{i}\cdot C_{o}$	PS·H·W
Sep_7 × 7	$C_{i}$	$C_{o}$	S	3	$C_{i}$	1	ReLU	7 × 7	$49\cdot C_{i}+C_{i}\cdot C_{o}$	PS·H·W
Dil_3 × 3	$C_{i}$	$C_{o}$	S	2	$C_{i}$	2	ReLU	3 × 3	$9\cdot C_{i}+C_{i}\cdot C_{o}$	PS·H·W
Dil_5 × 5	$C_{i}$	$C_{o}$	S	4	$C_{i}$	2	ReLU	5 × 5	$25\cdot C_{i}+C_{i}\cdot C_{o}$	PS·H·W
C_3 × 3_g	$C_{i}$	$\frac{C_{o}}{2}$	S	1	g	1	CReLU	3 × 3	$\frac{9\cdot C_{i}\cdot C_{o}}{2\cdot g}$	PS·H·W
Fire	$C_{i}$	$C_{o}$	S	1	1	1	ReLU	3 × 3	$\frac{C_{i}^{2}}{4}+\frac{5\cdot C_{i}\cdot C_{o}}{4}$	PS·H·W
Sep_res_3 × 3_g	$C_{i}$	$C_{o}$	S	1	g	1	ReLU	3 × 3	$\frac{C_{i}^{2}+C_{i}\cdot C_{o}}{g}+18\cdot C_{i}$	PS·H·W
Sep_res_5 × 5_g	$C_{i}$	$C_{o}$	S	1	g	1	ReLU	5 × 5	$\frac{C_{i}^{2}+C_{i}\cdot C_{o}}{g}+50\cdot C_{i}$	PS·H·W
Dil_res_3 × 3_g	$C_{i}$	$C_{o}$	S	2	g	2	ReLU	3 × 3	$\frac{C_{i}^{2}+C_{i}\cdot C_{o}}{g}+18\cdot C_{i}$	PS·H·W
Dil_res_5 × 5_g	$C_{i}$	$C_{o}$	S	1	g	1	ReLU	5 × 5	$\frac{C_{i}^{2}+C_{i}\cdot C_{o}}{g}+50\cdot C_{i}$	PS·H·W

## Appendix B. More Detailed Experimental Results

In this section, the lightweight architectures of VGG16, DenseNet121 and MobileNetV2 after being compressed are presented in detail.

## Appendix C. Different Sets of Operators in the Branches of Reconstructed CNN (SOP)

**Table A2:** Different sets of operators in the branches of reconstructed CNN.

SOP1				
N_3 × 3	N_3 × 3_2	N_3 × 3_4	N_3 × 3_8	Sep_3 × 3
C_3 × 3	C_3 × 3_2	C_3 × 3_4	Dil_5 × 5	Sep_5 × 5
SOP2				
N_3 × 3	N_3 × 3_2	N_3 × 3_4	Sep_5 × 5	Dil_3 × 3
N_3 × 3_8	N_3 × 3_16	Sep_3 × 3	Sep_7x7	Dil_5 × 5
SOP3				
N_3 × 3	N_3 × 3_2	N_3 × 3_4	Sep_res_3 × 3	Sep_3 × 3
Fire	C_3 × 3	C_3 × 3_2	Sep_res_5 × 5	Sep_5 × 5
SOP4				
Fire	C_3 × 3_2	Sep_res_3 × 3_4	Sep_res_5 × 5_4	
C_3 × 3_4	N_3 × 3	Dil_res_3 × 3_4	Dil_res_5 × 5_4	
SOP5				
N_3 × 3	N_3 × 3_2	N_3 × 3_4	Sep_res_3 × 3	Dil_res_3 × 3
Fire	C_3 × 3	C_3 × 3_2	Sep_res_5 × 5	Dil_res_5 × 5
SOP6				
Fire	C_3 × 3_16	Sep_res_5 × 5_8	Sep_5 × 5_8	
C_3 × 3_8	N_3 × 3_16	Dil_res_5 × 5_8	Dil_5 × 5_8	
SOP7				
N_3 × 3	N_3 × 3_2	N_3 × 3_4	Dil_res_5 × 5	
Fire	C_3 × 3	C_3 × 3_2	Sep_res_5 × 5	
SOP8				
N_3 × 3	N_3 × 3_2	N_3 × 3_4	Dil_res_5 × 5_8	
Fire	C_3 × 3_4	C_3 × 3_8	Sep_res_5 × 5_8	
SOP9				
N_3 × 3	N_3 × 3_2	N_3 × 3_4	Dil_3 × 3	
Fire	Dil_5 × 5	Sep_3 × 3	Sep_5 × 5	
SOP10				
N_1 × 1	N_1 × 1_2	N_1 × 1_4	N_1 × 1_8	
C_3 × 3_4	N_3 × 3_4	N_3 × 3_8	Fire	
